# Extracts and Gleanings

**Published:** 1876-12

**Authors:** 


### Extracts and Gleanings.

---

*Bronchial Catarrh.*—The child, its mother says, is four years old, and does not present the appearance of being much diseased. Its face is rather bright, its body is well nourished, its chest-walls look quite natural, its breathing is not labored. Percussion gives uniform sounds on either side, and discovers no dullness. This is an important point. It indicates that the parenchyma of the lung is not involved; that there is no solidification of pneumonia present. Whatever trouble it has must be confined to the larger tubes. On auscultation you will note sounds like those produced by blowing soap-suds through a pipe. This is caused by the air rushing through the mucus gathered in the larger tubes. These sounds are distributed generally over the chest, the trouble is bi-lateral, as it is called, and the rales are best heard behind, where the larger bronchial tubes are situated. The undue quantity of mucus is a foreign body in the lung, and excites the cough. There is no fever. We have here a simple bronchial catarrh—or subacute bronchitis, as it is called—not apt to be dangerous unless occurring in the very young or very old, when the accumulation of mucus may at times stop the ingress of the air. Our remedy in these cases, when the trouble persists and demands interference, is chiefly the muriate of ammonia. As you will see a great deal of this prescribed at the clinic, I may tell you at the outset how it acts. It belongs to the class of stimulating expectorants which cause an increased flow of mucus, and would hardly seem indicated here. But muriate of ammonia produces a rapid exfoliation of diseased epithelium of mucus membrane, and cleanses the part; and from this action we expect it to do good in the present case. Its use should not be too prolonged, otherwise it may itself set up diseased action. The dose for adults is from five to twenty grains; for children, two to five grains. Our recipe will be

R. Muriate of ammonia..............................dr. i;
Extract of licorice..................................gr. x;
Syrup of wild cherry..............................oz. i;
Syrup of ipecac.....................................dr. ss;
Water...................................................oz. ss. M.

S. A teaspoonful three or four times a day.

—Dr. Palmer, in *Louisville Medical News*.

*Remedy for Dysmenorrhœa and Consequent Sterility.*—The following was published in the New Orleans *Medical News* nearly twenty years ago; it is republished at the request of a physician who has used it successfully in his practice: Dr. E. D. Fenner states that he has used for some years, in the treatment of dysmenorrhœa, with great success, the following mixture, originally recommended by Dr. Falk, of London: Take of gum guaiac, 1 ounce; balsam canadense, 1 ounce; ol. sassafras, 2 drams; merc. corrosiv. sublimat. 1 scruple; rect. spt. vini (alcohol,) 8 ounces. "Dissolve the guaiac and balsam in one half the spirit, and the corrosive sublimate in the other. Let the guaiac and balsam digest for several days; then pour off the clear liquor, mix with the sublimate, and add the oil. Dose—ten or twenty drops night and morning in a glass of wine or water, *pro re nata.*" This was called by Dr. Falk "*Tinctura Antacrida.*"

Dr. Fenner says that he usually directs the patient to begin a day or two before the expected period and take twenty-five drops in an infusion of sage or sweetened water, night and morning, until the discharge is freely established; then cease till the next period. In obstinate and severe cases the medicine should be commenced a week or ten days before the period, and if the pain appears the medicine should be taken every four or six hours till relieved. The pain usually disappears as soon as the discharge becomes free, but in most cases the discharge comes on without pain after taking a few doses. "I have known immediate relief to be given by a single dose taken in the paroxysm; but I have seen cases in which the pain was excruciating, causing shrieks and even violent convulsions. In such I have had to resort to a more prompt and efficient anæsthetic, as the inhalation of chloroform, or the following, which I have often known to act like a charm: Take of spirit camphor, 3 drams; chloroform, 2 drams; tinct. opii, 1 dram; mix; dose—a teaspoonful in sweetened water once an hour till relieved. In violent hysterical spasms there is nothing comparable to the inhalation of chloroform. In the treatment of dysmenorrhœa it is important to obviate costiveness by the use of aloetic pills. When dysmenorrhœa is relieved by this treatment conception almost invariably soon occurs in married women."

*Picric Acid for Sore Nipples.*—In an article published in the *Courrier Medical*, Dr. Charrier recommends the use of picric acid in solution as a cure for sore nipples. The application has the effect of rapidly removing the pain, and checking the morbid secretions. The acid is used in the following manner; It is important to employ the chemically pure product, perfectly free from soda, and two solutions of different strength are prepared. The first, or *concentrated*, solution is made of three drachms of picric acid dissolved in two pints of water; and the second, or *weak*, solution only contains sixteen grains of acid to the same quantity of water.

To apply the lotions, the nipple is first well cleaned and washed with a fine sponge and lukewarm water; then a very fine camel's-hair pencil, dipped into the concentrated solution, is gently passed over the chapped and inflamed parts. This is done once a day, in the morning; and each time after the child has been suckled, the nipple is immersed during three or four minutes in a small glass filled with the weak solution.

After from twelve to twenty-four hours, the sharp pains experienced when the child is suckled begin to disappear, and all the red and inflamed parts assume again their natural color. The picric acid solution extinguishes, as it were, the inflammation on the spot, prevents the lymphangitis from extending further, and therefore averts phlegmons and abscesses of the mammæ. Another advantage of the acid is that it tans the thin epidermis of the breast, rendering it thereby less liable to alterations.

*Nitrite of Amyl.*—This agent appears to be growing into favor among practitioners on both sides of the Atlantic. It is employed to a considerable extent in the asylums of America in the treatment of the epileptic insane. In nearly all the cases where the paroxysms are foreshadowed by an aura or any other premonition, it is effectual in preventing the attack. The epileptic wards are described as having been "revolutionized" through this agency. It has been used in asthma, angina pectoris, and dyspnœa from various causes. There appears to be very little danger from it under any ordinary circumstances. Anæmic persons require a larger quantity, and should use it with more care. Dr. Squibb says he was exposed to its vapor to an unusual extent in consequence of the breakage of a pint bottle, and the necessity to remain and exert himself in an atmosphere heavily charged with the vapor, in order to prevent an explosion, which was imminent. The feeling of swelling of the head and body, and a sensation as if about to fall, were extreme for a time, but passed off without ill effects. The sensorium was clear during the whole time. Nitrite of amyl is administered in the same manner as chloroform. The most popular method is to inhale from a small vial, the mouth of which is applied to one nostril. One to three full inspirations are sufficient. Asthmatics and others often carry it in the vest-pocket to use in emergencies. It requires a glass stopper.—*Pacific Med. and Surg. Journal.*

*Treatment of Prolapsus Recti.*—In a letter written shortly before his death, Professor Stromeyer said: "Among the presents which I received there was one, however, which, a trifle in itself, was highly valuable to me, because I could regard it as a proof that my physiological way of explaining cases is still successful in practice. It was a small piece of carpet-work, made by a young lady whom I had lately cured of a large prolapsus recti by warm baths and a little magnesia. I consider this dreadful complaint as a consequence, not of paralysis, but of irritation—spasmodic reflex action. The case would have been operated upon, probably, by actual cautery, if my interference had not taken place.

*Treatment of Angina Pectoris.*—Prof. See, of Paris, says this affection is not a neurosis, but an ischæmia combined with pain. The treatment should, therefore, be twofold. As the pain, which by its violence can stop the breathing, can kill the patient in a few minutes, it is to that we must first address ourselves when called during an attack. To this end morphia hypodermically is the best, and the administration of it in this way should be continued at least twice in the twenty-four hours, until the attack has completely disappeared. The morphia not only acts by suppressing the pain, but it assists the circulation also, and thus directly addresses itself to the ischæmia, which stops the heart from receiving sufficient blood, causing it to lessen. Together with morphia injections, enemata of chloral should be given to the extent of two to three grammes. While advising chloral, Professor See cautions against the use of chloroform, which ought not to be used, owing to its tendency to paralyze the heart. Nitrite of amyl has no action as a sedative, but its effect to produce dilatation of the vessels may render it useful. Belladonna produces no effect, and the use of anti-spasmodics in a disease of such severity is absurd. Acetate of ammonia has a certain value as an excitant of the circulation, and because it acts on respiration, but it is inferior in value to morphia. Although prejudice may sometimes render it desirable to use cuppings, friction and heat, they are really of no service. During an attack the use of the bromides is inadmissible, because they produce contraction of the blood-vessels instead of dilating them; but, like digitalis, they may be valuable during the intervals as regulators of the circulation. Arsenic, however much vaunted, does no good. Hydrotherapy is highly dangerous, either a return of the attack or cerebral congestion being results to be feared from its employment.—*Paris Medical.*

*Treatment of Syphilitic Buboes by the Parenchymatous Injection of Iodide of Potassium.*—Dr. Jacubowitz, of Nagy Karoly, (*Der Praktische Arzt*, No. 7, Jahrgang xvi.) starts from the principle that no inflammation to which a degenerative action is attributable is occasioned by the injections, but that by this means a solvent of a non-irritating character is brought into direct contact with the glandular tissue. He avoids tincture of iodine, all alcoholic fluids and carbolic acid, and uses instead a weak solution of iodide of potassium in the proportion of about one part to thirty of water. He gives two cases in which he obtained extraordinarily successful results. In one case he made a puncture into the most prominent part of a gland which was enlarged to the size of a goose's egg, pushing the needle in obliquely to a considerable distance. After injecting about the fourth of the syringeful a resistance was felt; he then withdrew the needle for a short distance, penetrated a septum on one side, and again injected a quarter part. By repeating this process he threw in about fifteen grains of the iodide in one ounce ot water. The tumor almost immediately became harder, smaller, and less painful. After four injections performed in the course of two days the tumor gradually dwindled to the size of a hazlenut, and ultimately vanished altogether. The second case was very similar. Here, however, two dark-blue bodies remained, which were so hard that it seemed to be impossible to inject them. Dr. Jacubowitz, however, injected hypodermically the iodide on two occasions, and with perfect success. Ten injections were required altogether. The small quantity of the iodide required to produce the effects observed is very remarkable.—*Practitioner*, June, 1876.

*On Juniper Fumigations in the Treatment of Skin Diseases.*—Dr. Cæsar Boeck (*Vierteljahesschrift fur Dermatologie und Syphilis*, 1875, 4 Heft) describes the following mode of treatment: The surface of the body is subjected to the action of the fumes of moist juniper leaves in cases of prurigo, uticaria, and pruritus cutaneus, with great mitigation of the itching. For example, a woman fifty years of age had been tormented with urticaria from March to September, and was obliged to scratch herself for about an hour every evening before going to sleep. The usual remedies had been fruitless. After two fumigations she was better; after ten (one daily) she left off scratching, and no more fumigations were required.—*London Medical Record*, May 15, 1876.

*Injection of Quinine in Gonorrhœa.*—Radha Nauth Roy, Assist. Surg., extols (*Indian Med. Gazette*, May, 1876) the efficacy of injections of quinia in gonorrhœa. He states: "I was once tempted to try it in a case of acute gonorrhœa, where scalding was unbearable, and discharge profuse, and to my utter surprise after the third day I found the man quite relieved. He described to me the soothing effect of the injeetion as something cold like ice. The discharge was so much diminished that his clothes were scarcely stained after the third day. There was no more incessant desire to void the urine, and he was to all appearances comfortable. My success in this case made me bold enough to use it in other cases, and I have invariably found the disease yield both in its acute and chronic stage under its influence. It acts as a tonic and astringent to the mucous membrane of the urethra. I have also used it in some cases of cystitis with much benefit. I generally use it dissolved in sulphuric acid dil. mixed with rose-water. Two grains of quinine sulph. dissolved in acid. sulph. dil. *m* viij. or *m* x, and mixed with an ounce of rose-water—to be used twice for injection. At the same time I give copabia mixture to my patients. In almost all the cases I have found it act like a charm. The disease is generally cured within a week, but chronic cases take a longer time In a few acute cases it took more than a fortnight, but the delay in them was attributable to their irregular habits during this treatment."

*The Treatment of Enuresis.*—Dr. Ludwig Fleischmann speaks (*Oesterrisch Jahrb. fur Pœdiat.*) in very high terms of the results of the treatment of incontinence of urine in children by faradization, of which he has made trial in a large number of cases. One pole of the battery is introduced into the rectum of boys, or the the vagina in girls, and the other is applied over the pubes. A very gentle current is used at first, and it is employed every second or third day. It has been found that the attempt to apply electricity more directly to the neck of the bladder by introducing a sound into the urethra does injury by the production of cystitis. Dr. Fleischmann's patients all received benefit from the treatment, and some were cured after only three or four sittings. He finds, however, that all varieties of the complaint are not equally suitable for the method. It is more especially advantageous for those cases in which the fault is due to weakness of the sphincter, and particularly those in which there is incontinence by day as well as by night. When the complaint appears to be due to excessive irritability of the neck of the bladder, without any muscular weakness, the patients receive some benefit, but are more liable to relapse. More permanent good is then obtained by the use of belladonna.—*Obstetrical Journal of Great Britain*, May, 1876.

*Case of Tapeworm Successfully Treated.* By Miles J. Birdsong, M.D., of Carthage, Texas.

Mrs. H., aged 29 years. The mother of five children, the youngest four months old, her general health good with good flesh. Nothing strange about her appearance or appetite. But has been occasionally afflicted, for two years, with pains in her abdomen, which she called colic, brought on from slight exposure to cold or from taking something cold on her stomach. On June the 6th, my attention was called to the case by her husband presenting a portion of the worm for inspection. Prescribed calomel and santonin, xv grs. each, to be taken at bedtime, to be followed with castor oil and sp. turpentine, next morning, discharged ten feet of worm.

*June* 9. Prescribed oleum terebinth fl. 2 drams, to be taken at bedtime with castor oil, next morning discharged ten or twelve feet more worm.

18*th.* Prescribed decoction pomegranate root, prepared after the formula in United States Dispensatory, one ounce to be taken every hour for eight consecutive hours, after fasting twenty-four hours. The remainder of the worm was discharged during the day while taking the decoction. The whole length of worm discharged was forty-five feet, according to the best estimate we could make.

*The* taste of quinine is admirably disguised by the Aromatic Elixir of Licorice. It removes in a great measure the difficulty of administering this drug to children

*Tape Worm.*—Dr. F. A. Ross presented a specimen of tape worm, which had been passed by one of his patients—a midle-aged gentleman. The Doctor. convinced of the presence of the worm in the intestinal tract, had recourse to a prescription, the formula of which came into his possession some years since. The medical gentleman who used it and wrote the formula, gave it his highest commendation, claiming that it would bring away the entire worm in less than six hours. Dr. Ross had only had the opportunity of testing the remedy in one case prior to the one now under consideration. In the first case (a negro woman), the entire worm was expelled in a little more than six hours; in the case of the gentleman furnishing the specimen here presented, less than six hours was required. The formula for the prescription is as follows:

R. Pomegranite root..................................oz. ss.
Pumpkin seed.........................................dr. ss.
Ext. male fern........................................dr. j.
Powd. ergot...........................................dr. j.
Powd. gum acacia.....................................dr. ij.
Croton oil...............................................gtt. ij.

The pomegranite root and pumpkin seed are to be thoroughly bruised, and with the ergot, to be boiled fifteen minutes in 8 oz. of water, and strain—the croton oil to be rubbed up with the acacia and extract filix mas and formed into an emulsion with the decoction.

S. To be taken in one dose after fasting.—Mobile Medical Society, *Virginia Medical Monthly.*

*Solution of Salicylic Acid.*—Emlen Painter, Ph.D., (*Pacific Med. and Surg. Jour.*, April, 1876,) suggests the following formula, which contains two grains of the acid to the fluiddrachm:

Acid. salicylic..................................gr. xxxii;
Sol. acet. ammon..................................oz. ii.

Dose—A teaspoonful, to be increased or diminished as required.

*Mist. Asthmatica. B. & C. H.*—

R. Spts. Æth. Co........................ } aa oz. 1

Sol. Morph. U. S..................... }

M. Tablespoonful.

*Nitrate of Lead as a Local Agent in Erysipelas.*—John Firnat, M.D., reports cases and says: "After having tried tincture of iodine, camphorated ether, nitrate of silver, carbolic acid with oil of turpentine, acetate of lead ointment, oxide of zinc ointment, and styptic collodion, in erysipelas, in connection with internal treatment, I have found nitrate of lead with glycerine to give far more satisfaction. . . . . I have also used the nitrate of lead, 10 to 15 grains to oz. j of glycerine, in eczema of the head, washing previously with tar soap, and then applying it with entire success, in connection with small doses of Fowler's solution and iodide of potassium."—*Medical Times.*

R. Strychinæ sulph............................gr. j.
Cupri sulph....................................gr. ij.
Cinchoniæ sulph ..............................drs. j.
Ferri sulph.....................................drs. iss.
Acid. sulph aromat...........................q. s.
Aquæ..............................................oz. iv.

M. Ft. sol. S. A teaspoonful three times a day after meals. Used as tonic, especially in obstinate Intermittent.

(University Dispensary Tonic.)

—*Louisville Medical News.*

*To Check Colliquative Sweating.*—The exhaustive sweats in surgical diseases and phthisis are entirely controlled, according to Dr. Thomas J. Dunott, of Harrisburg, Penn., by small hypodermic injections of atropiæ and sponging with hot vinegar. In in a case in point, given in the *Virginia Medical Monthly*, he writes, of a case of osseous injury: "He sweats profusely and constantly; to have ice pills and hypodermic injection of one hundredth of a grain of atropiæ sulph.; also to be sponged with hot vinegar. This controlled the sweating, which was so profuse as to keep the bed-clothing saturated whenever the atropia and sponging were omitted. It is my belief that a very small dose of atropia, when combined with the hot-vinegar application, will be most effective in controlling this exhausting discharge from the skin. Neither used alone would be successful; but my experience with atropia is limited to doses no larger than the one mentioned—one hundredth of a grain.—*Med. and Surg. Rep.*

*Poison-Oak Eruption.*—Dr. Bernard (*Louisville Med. News*) says: "I think I can still add one to the list of reliable remedies for the torment inflicted by that infernal thing, to wit: fluid extract of gelsem. semperuirens, applied by simply brushingit over the affected part with a feather.

"Some few weeks ago I had to prescribe for a boy who had got the poison, of all places in the world, all over the genital organs. The burning sensation invariably came on about sunset, and tormented the patient all night long, subsiding always at daylight. (Here was a good hint to try quinine, but I failed to use it.) After trying about a dozen of so-styled 'infalliables,' I heard of the gelseminum, and concluded to try it; for I was just then 'at the end of my row,' and badly bothered to find a remedy. The first application gave complete relief, and the patient slept soundly all night. After that the gelseminum was applied three more times, and that ended the job; and a devilish troublesome job it was until the gelseminum let me out of the scrape.

"Some thirty years ago I saw sulphate of copper (two grains to one ounce of water) used in a great number of cases, and with general success; but lately I have known it to fail several times. Should I ever be called upon to treat another case, I would certainly try quinine at the start."

*Treatment of Chronic Diseases of the Stomach by Alkaline Injections.*—Dr. James H. Hutchinson reported to the Pathological Society of Philadelphia (*Med. Times*, May 27, 1876) a case of cancer of the pylorus, in which great relief followed the washing out of the stomach on alternate days with dilute alkaline solution (a drachm of bicarbonate of soda to a quart of water) as recommended by Kussmaul in 1867.

The following conclusions seem to be deducible from Dr. Hutchinson's case:

1. That washing out the stomach will be useful in dilatation of that organ dependent upon stricture of the pylorus, even if this be due to malignant disease, by lessening the frequency of the vomiting.

2. That it diminishes the intensity of the pain, by preve[illegible]ng extreme distention of the stomach, and by the removal of its irritating contents.

3. That it renders possible the introduction of food into the stomach, and its digestion.

4. That it may sometimes facilitate diagnosis.

*Treatment of Polyuria.*—M. Hayem mentioned at a meeting of the Paris Biological Society, the case of a man aged forty-eight, who had enjoyed good health up to 1867, when he suffered from a nervous attack, loss of consciousness, and paralysis of the right arm. In 1874 the sight became impaired. The patient remained in good health up to February, 1875, when he became affected with polyuria, polyphagia, and polydipsia, soon connected with nocturnal incontinence of urine. He was ordered issues to the lumbar region, and iodide of starch. When the patient was first seen by M. Hayem the face was puffed and the lower limbs œdematous. The urine did not contain any albumen, but a certain amount of sugar. He voided in the twenty-four hours four quarts and a half of urine, half an ounce of urea per quart—viz: about two ounces altogether. After a treatment of twenty-eight days, the patient taking about one grain of opium per diem, the quantity of urine diminished to about three pints per day, and the urea to less than half an ounce per quart—viz: seven drachms in the twenty-four hours. The general health was much improved.

*Treatment of Diphtheria.*—Dr. Cesare Ciattaglia gives an instructive communication on the cure of diphtheria in the *Gazetta Medica di Roma*, which is abstracted in the *Lancet.* For some time he has been successful in treating it with the chlorate of potash internally and the application of the hydrate of chloral to the false membranes. With these he combines a tonic and restorative diet. To children of 3–6 years of age he administers the chlorate of potash in doses varying from 10–15 grammes a day dissolved in 140 of water; while the hydrate of chloral, in the proportion of 4 grammes of the hydrate dissolved in 20 grammes of glycerine is painted over the diphtheritic patches three or four times a day. For adults the dose of the chlorate of potash is 20 grammes (300 grains). Dr. Ciattaglia points out the certainty with which the application of glycerine solution of hydrate of chloral arrests the progress of the formation of the false membranes. He disclaims any pretension to originality in the nature of the above remedies, since the chlorate of potash was introduced by Vogel, and Ferrini suggested the use of the hydrate of chloral dissolved in glycerine.—*Lancet.*

*On the Treatment of Chorea*.—M. Fabry relates in the *Bulletin de Therapeutique* (quoted in *Paris Medical*, March 9, 1876) some observations carried out in the service of Dr. Perroud, of Lyons, on the treatment of chorea by ether-spray. This therapeutic agent, employed for the first time in 1866 by Lubetski, has given good results in Dr. Perroud's hands.

Applications of ether-spray are made along the spine by some spray-producing apparatus, such as those of Richardson or Marinier. Each application lasts from four to eight minutes. At the commencement of the treatment applications should be made three times a day; afterwards the number may be reduced to two.

Ice produces the same effect as ether-spray; a piece of ice may be passed along the length of the vertebral column for five minutes at a time.

These two means have effect by their refrigerant revulsive action on the excito-motor point of the nervous centres.—*Lond. Med. Record*, May 15, 1876.

*Treatment of Fissured and Ulcerated Nipples.*—In the *Annales de Gynecologie* Dr. Legroux advises the following treatment: Spread with a camel's-hair brush a layer of elastic collodion around the nipple, in the radius of an inch or more; a piece of gold-beaters' skin should then be placed over the nipple and collodion, taking care to make a few holes with a pin over the part of the gold-beaters' skin which covers the nipple, so as to allow the milk to ooze through. No collodion should be spread on the nipple itself, as more pain might thereby be occasioned. By the rapid evaporation of the ether the collodion dries up and the gold-beaters' skin adheres. The nipple is thus more or less pressed down by the latter, which in drying becomes tense. When the child is to be nursed the end of the nipple should be wetted with a little water. The covering of gold-beaters' skin becomes soft and supple, and allows the child to suck without distressing the mother.—*Med. and Surg. Rep.*

*Injections of Chloral in Hydrophobia.*—M. Prevost reported the following case at the seance of the Societe de Biologie, on June 3d: A woman was bitten by a rabid cat in the month of July, 1875. The wound was cauterized with ammonia, but no other treatment was adopted. The patient became very melancholy, and was pursued by a presentiment that she was about to become mad, but her general health continued good and the wound healed rapidly. Six weeks after the injury she began to suffer again from pains at the seat of the injury, and suppuration set in. She became nervous and irritable, and the presentiments were redoubled, and a few days later she was seized with a perfectly characterized attack of rabies. The paroxysms were extremely violent and very numerous. Hypodermic injections of morphine and inhalations of chloroform were tried, but failed to quiet the patient. Injections of chloral into the dorsalis pedis vein were then tried; one drachm was injected the first time, and the injections were repeated several times in twenty-four hours. These quieted the patient and produced a pretty calm sleep, but she gradually became weaker and died; without, however, having any more paroxysms. At the autopsy only the cord could be examined, and it was found to be healthy.

M. Hanot stated that he had used this treatment in a case of hydrophobia in 1874, and his patient was quieted for twenty-four hours.—*Gazette Hebdom. de Med. et de Chir.*, June 9th.

*To Prevent Troublesome Perspiration of the Feet.*—Dr. I. H. Crawford, of Wicasset (State not mentioned), writes to us that in connection with the use of cold water and soap, the following formula has, in his experience, relieved this disagreeable affection:

Salicylic acid.................................gr. i.–ij.
Lycopodium powder..........................oz. i.

M. To be dusted on the feet.—*New Remedies*

*Sulphate of Cinchonidia.*—Dr. Bensley, one of a committee appointed by the British East India Government to test the value of the cheap alkaloids of cinchona bark, says of sulphate of cinchonidia, it is admirably adapted to those requiring a tonic febrifuge, in which there is at the time a great tendency to diarrhœa' or where diarrhœa already exists; but where quinine produces these disturbances, the cinchonidia is well borne. None the less valuable is it in consequence of the mildness of its influence on the nervous system. He further says: I have used it extensively in the fevers of children on account of its mildness, and because it is less liable to produce head and bowel disturbances than the other alkaloids.

Dr. Campton, Ky., says in a paper on this remedy: Upwards of thirty of my cases were children, varying in age from one to nine years. I have such confidence in it that it is the only preparation I prescribe for children. It is a well-known fact that there exists with many persons a strong prejudice against quinine, and it is a great advantage to be able to say to such persons that you have a remedy that will be equally efficient, in all cases where quinine is indicated, without being liable to the objectionable effects of that remedy. The advantages to be derived from the use of sulphate of cinchonidia may be summed up as follows: Fewer relapses follow its administration. It is better tolerated by the stomach, not being nearly so liable to produce nausea and vomiting. It does not create the same amount of ringing and noise in the ears that characterizes quininism. It is not liable to produce temporary deafness. It does not produce the nervous excitability. It does not increase or produce diarrhœa. It obviates the prejudice existing against quinine. Its cost is but one-third that of quinine.

*Lemon Tonic. Ch. Hospital.*—

| | | | |
|---|---|---|---|
| R. | Tr. Ferri Chlor | fl. dr. | ½ |
| | Cinch. Sol. Sulph, [grs. 30 in fl. oz. 1] | fl. oz. | 1 |
| | Acid. Citric | dr. | ½ |
| | Syrup. Simpl | fl. oz. | 1½ |
| | Aquæ | q. s. ad fl. oz. | 4 |

M. Tablespoonful.

*How to Decide the Question of Operation in a Case of Peri-typhlitis.*—The case was one in which well marked symptoms of peri-typhlitis had been developed, and a hard mass extending from low down in the iliac fossa to above the crest of the ilium could be distinctly mapped out by palpation and percussion. Tenderness was very well developed upon pressure over the same region, and there was arrest of respiratory motion below the umbilicus. Pulse 110, and temperature 101½° F. The patient received a moderate amount of opium, was kept perfectly quiet in bed, and had local applications of light warm poultices. At the time of our visit he had been in the hospital three days, and had been sick a week before his admission. On the seventeenth day of his sickness there was less pain, the tumor had diminished in size, and it was quite evident that resolution was taking place. That fact led the visiting physician to remark that he had seen several cases of peri-typhlitis which had progressed until it had seemed that suppuration was inevitable, and yet from that point a change for the better had occurred, and resolution had taken place. He regarded it as a matter of great difficulty, in many of these cases at least, to determine the exact time when pus had been formed, and in no case, therefore, would he consent to any surgical operation for the evacuation of an abscess in that region until pus had been detected by means of the aspirator. Under such circumstances the aspirator was regarded as an instrument that could render signal service, and when pus could be reached by the use of the needle, then, and not until then, should recourse be had to any surgical operation of greater severity. In the case before us a good recovery took place.—*Med. Record.*

*Stokes' Expectorant Mixture.*—

| | | |
|---|---|---|
| R. | Ammon. carb | gr. 32 |
| | Ext. fl. senegæ<br>Ext. fl. scillæ | aa fl dr. 1 |
| | Tr. opii camph | fl. dr. 6 |
| | Aquæ | fl. oz. ½ |
| | Syrup. tolut | q. s. ad fl. oz. 4 |

M. Teaspoonful.

*Capillary Puncture of the Intestines in Tympanites.*—The *Bulletin Medical du Nord* contains a paper by Dr. Cuïgnet, from which we extract the following: "1. The puncture should be made by giving a rotary motion to the needle, which is held between the fingers at the surface of the body; 2. It can be perceived the moment the needle reaches the gaseous cavity, as well as the moment it touches the opposite wall, thus showing the exact dimensions of the cavity; 3. The gas does not escape spontaneously, however distended the cavity may be which contains it, but it must be withdrawn by aspiration; 4. Only the fold of intestine in the immediate vicinity of the puncture is evacuated, but all of the folds of the intestine must be punctured to obtain any considerable relaxation; 5. Each fold as it is punctured collapses, and its place is filled by the two folds above and below it, which maintain the tympanites in the same region until they are also punctured; 6. Either the gas alone may be withdrawn, or both the gas and the liquid matter n the intestine, by graduating the depths to which the needle is made to penetrate; 7. It is esteemed prudent to always extract the liquid in the vicinity of the puncture."

*Topical Treatment of Chrhonic Dysentery.*—The reporter narrates three cases of topical treatment of dysentery, followed by cures. They were of several months standing each. The first case, that of a girl fourteen years old, was of six months duration. She was in a very low condition, with a pulse 130, and scarcely perceptible, skin covered with a clammy sweat. Her body had emitted a cadaveric odor for several days; death seemed inevitable. After etherization, a bivalve speculum was introduced into the rectum, and the "mucous membrane was found highly inflamed and studded over with small yellowish ulcers, which, on slight pressure, emitted a colored fluid." Silver nitrate was freely applied to every part of the bowel, as high up as could be reached with the aid of a retractor. This operation was followed by an ability to control the bowel. The appetite was improved, strength increased, and recovery of the vital parts was very speedy. Daily injections of carbolic acid solution (one part to eight of water) were used. In two weeks the patient made a complete recovery. The other two cases were similarly treated and recovery followed.—*N. Y. Med. Journal.*

*Chloral as an Application to Ulcers.*—Mr. Lucas, at Guy's Hospital, treats ulcers with a solution of four grains of hydrate of chloral in an ounce of water. The application of a lotion of this strength is often attended with considerable smarting, which may last a quarter of an hour; but the smarting becomes less at each subsequent application. In cases where the patients have complained much of the smarting the lotion has been diluted to the proportion of three or two grains to the ounce The treatment of foul sloughing ulcers by means of chloral lotion has been attended with great success, the surface of the sore quickly cleansing and assuming a healthy appearance, while the subsequent healing has advanced with a rapidity in some cases quite astonishing. Under the use of chloral lotion the ulcers quickly became sweet and clean, and the cuticle spread over them with vety great rapidity, even while the surfaces of the sores were still considerably below the level of the surrounding skin.—*Lancet.*

*Ulceration of the Frœnum Linguœ in Whooping-Cough.*—Some discussion has been raised by Dr. Morton's paper on the above subject, read at the Harveian Society. The coincidence of ulceration in this particular position with pertussis is not new, though English authors have not referred to it, except casually, in association with stomatitis. This ulceration has been described in both French and German literature, more especially by Bouchut in his works on diseases of children and new-born infants, though what relationship it has to pertussis, or why it exists at all in that position, is not decided. To Dr. Morton, however, is due the credit of collecting statistics of the per centage of cases of whooping-cough in which it occurs, and also of bringing it prominently forward for the consideration of English observers.—*Med. Press & Circular.*

*Restoration of Milk-Supply.*—Dr. Bizzell reported a case of stoppage in the flow of milk in one breast, on account of a cicatricial deformity of the nipple preventing the outflow. He concluded to try the effect of adhesive strips, which were applied in such a way that they supported the organ, and at the same time exerted a firm pressure upon the organ. The pain and uneasiness, which had been considerable, ceased immediately, and did not return. The engorgement of the breast so much abated that in thirty-six hours the straps were quite loose; they were then removed and fresh ones applied, which were suffered to remain on for four or five days. On removing the straps, the breast was soft and flaccid; no more milk was poured out, nor any further trouble experienced—the other breast furnishing milk in abundance.—Mobile Medical Society, *Virginia Med. Monthly.*

*Poisoning by Oil of Red Cedar* (*The Detroit Review of Medicine and Pharmacy,* July, 1876.)—Dr. D. C. Holley reports the case of a woman who took half an ounce of red cedar oil—*Ol. Juniperi Virginianæ*—for the purpose of producing abortion. In a few moments she had symptoms of congestion of the brain, and soon became comatose. Violent convulsions came on, and occurred in rapid succession. Emesis was produced by sulphate of zinc, chloral and bromide of potassium were freely given, and sinapisms were applied to the feet and epigastrium. She remained comatose for twelve hours, but then regained consciousness, and made a good recovery.

*Formation of Epidermis by the Transplanting of Hairs* (*Boston Medical and Surgical Journal,* June 1, 1876.)—Dr. Schweininger reports successful results in inducing cycatrization by transplanting to granulating surfaces hairs pulled out by the roots. Placed upon ulcers they formed as many centres of new epithelial growth, which spread outwards, coalesced, and produced rapid and complete cicatrization. These islands proceeded, without doubt, from the cells of the outer root-sheath, which is continuous with the epidermal cells of the rete mucosum, so that epithelium is here developed from pre-existing epithelial cells.

R. Ung. ox. zinc. ben..................................oz. iv;
Acidi carbolic...................................dr. ij.
M. Apply on cloths for Carbuncle.

—*Louisville Medical News.*

*On the Use of Xanthium Spinosum in the Treatment of Hydrophobia* (*Journal de Therapeutique*, No. 7, 1876 : Gozymala.)—G. is a physician in Podolia, where rabies frequently occurs in dogs and wolves, and where yearly a large number of persons are bitten by rabid animals.

For many years he has used the leaves of the xanthium spinosum in the treatment of this affection, and he thinks that from the results of his wide experienee he is justified in calling it an "infalliable" remedy. When the drug is administered early enough to those who have been bitten, the disease does not occur, while in persons who have been bitten by the same animals and have been treated by other methods, or not at all, hydrophobia is developed. The physiological action of the drug is similar to that of jaborandi, but far less marked.

It is administered in the form of powder to adults in doses of o. 3 gram. three times a day, while to children one-half the quantity is given, and the treatment should be continued about three weeks. G. is so fully convinced of the absolutely certain effect of the remedy that for a long time he has not cauterized the wounds due to the bites of rabid animals.

*Vomiting of Pregnancy Cured by Hyoscyamia* (*The Medical Press and Circular*, May 19, 1876.)—Dr. Pitois, Professor at the Medical school at Rennes, reports two striking cases of this. After trying, unsuccessfully, all the usual means, it occurred to him to administer a teaspoonful every hour of a mixture containing 5 milligrammes of hyoscyamia in 125 grammes of fluid. The next day the vomiting ceased, did not recur, and the patient went on favorably to the natural term of her pregnancy. A second case of the same kind was cured by the same remedy. Although hyoscyamia seems to have been successful in the treament of these two cases, its efficacy as a certain remedy for the vomiting of pregnancy is not by any means established. It is well that the drug should be used, that its value may be estimated; but, as the vomiting is due to what may be called natural causes, permanent relief can scarcely be expected to be obtained by the use of a drug.

*A New Appliance for Bloodless Operations.*—Mr. H. L. Browne, surgeon to the West Bromwich Hospital, proposes, in the *Lancet* for Jure 3d, a very useful modification of Esmarch's bandage. A suitable rubber ring is rolled along the limb and over a plug placed on the main artery. This plug is provided with a groove upon its upper surface, which receives the ring and keeps it from shifting. The rings are made of different sizes, as are also the plugs, although the latter are only used over the larger arteries. The apparatus may be used as an ordinary tourniquet, by stretching instead of rolling the ring over the limb and plug.—*Med. News.*

*Prevention of Miscarriage by Hypordermic Injections of Morphia.* Dr. A. B. Isham, of Cincinnati, reports (*Am 1. Jour. of Med. Science*) three cases of successful treatment by this plan, and holds that the manner of administration has been the cause of the small measures of success obtained by other practitioners; asserts that medicaments introduced into the rectum act in a very uncertain manner, and it is difficult to administer them by the mouth when the patient is vomiting, but the hypodermic injections obviate all these difficulties. He considers it useful in any case of threatened action.

*Fetid Feet.*—A very obstinate case of this complaint, in a workman, is reported in the *Bull. de. Ther.* by Dr. Ortega. In the manufactory in which he worked he was avoided by his fellow-workmen, and when he entered a room the window would be opened. He had consulted several physicians, but without success. The epidermis of the sole of the foot was white and macerated, and there were little ulcerations at the clefts of the toes and around the nails. M. Ortega advised him to apply compresses soaked with a solution of chloral, which had the effect of rapidly destroying the smell and curing the ulcerations.

*Mist. Bumstead.*—

| | | |
|---|---|---|
| R. | Bals. Copaiv............................ | fl. oz, ½ |
| | Tr. Ferri Mur................ } Tr. Canthrid. .................... } | aa dr. 2 |
| | Syr. Acaciæ....................... | q. s. ad fl. oz. 4 |

M. Tablespoonful (in gonorrhœa).

*Strong Solution of Salicylic Acid.—Formula of Solution.—*

Salicylic acid.. ......................................... 1 dr.
Potassium* acetate.. .......................................1 dr.

*Or Calcinm,—or Sodium,—or Ammonium acetate.

Glycerine......... .................................... .. .. 1 fl dr.
Water..........................................q. s. ad. 1 fl oz.

Mix, and dissolve by shaking. This solution contains one grain of salicylic acid in eight minims; but may be made even stronger, as the limit of solubility has not by any means been reached.

This solution has taken the place of all others in the practice in Bellevue and other hospitals, and from reports received from the physicians, it appears to act more rapidly and to be more easily borne than any other. In cases of acute rheumatism especially, has it been found very effective, much more so than the mixture formerly used of salicylic acid and bycarbonate of sodium.—*New Remedies.*

*Daturia as a Substitute for Atropia.*—It has been proposed to substitute daturia, the alkaloid of the Jamestown weed, for atropia, as a mydriatic. The two alkaloids were formerly considered as identical, but their difference is now acknowledged. Daturia is three times as active as atropia, and its dose is therefore much less. When applied to the eye it does not cause the pain and confusion of vision which follow the use of atropia. Its effects are more constant and persistent. Such are the statements ecently publishedɹ—*Pacific Med. and Surg. Journal.*

*Croton Oil Paint in Pelvic Cellulitis.*—Dr. Charles G. Budd (*American Journal Obstetrics*, April, 1876) says he has found the treatment of pelvic cellulitis by blisters, iodine and oleate of mercury very unsatisfactory Lately he has used, with happiest results, Corson's croton oil paint. R. Ol. croton dr. ij. ether sulph. fort., dr. iv; tinct. iodine, dr. ij; potass. iodide sc. j; iodine gr. x. Mix. In four cases, when a pelvic abscess seemed unavoidable, under the use of this paint the exudation fairly melted away.

*Right*—" All I have had to do I have done in kingly fashion I let tongues wag as they pleased; what I saw to be the right thing—that I did."—*Goethe.*

*Chronic Pneumonia of the Apex in Children.*—Dr. L. Fleishman (*Winer Med. Presse*, No. 20, 1876, *and Allgemeine medicinessche Central-Zeitung*, No 41) speaks of the frequency of this disease and the manifold difficulties attending an early accurate diagnosis. Such are: the small extent of the infiltration, which is limited to the apex, and is often to be recognized only after repeated careiul examinations; the frequent absence of marked physical signs, congh and sputa; and finally, the difficulty of examining a young, restless infant. The symptoms which we usually find in incipient phthisis in adults, such as cough, hæmoptysis, palpitations, anæmia and sinking in of the chest-wall, are absolutely wanting, or if present, we are then dealing with a long-standing infiltration, not with a commencing disease.

In observing teething children the author has noticed the following symptoms, which have led him to recognize or to suspect chronic pneumonia of the apex at an early stage.

(1.) One-sided swelling of the lymphatic glands of the throat, back of the neck or of the sub-maxillary region, when other local causes, such as pharyngitis, parotitis, alveolar inflammation, and diphtheria can be excluded, causes strong suspicion that there is pneumonia of the apex on the same side. The glandular swelling continues while the process in the lung is active, and ceases when the lung infiltration becomes stationary, the glands swelling and subsiding again with each advance of the inflammation. Such glands have been usually called scrofulous.

In enlargement of the glands before and behind the ear, the former is often due to inflammations of the eye, the latter to that of the ear.

Children over six years old do not show so marked a tendency to glandular enlargement.

(2.) Certain obstinate forms of conjunctivitis (scrofulosa), which in spite of all treatment and without apparent cause, return from time to time with great severity, if but one and always the same eye is attacked, point with great probability to disease of the lung of the same side.

(3.) Eczema of one-half of the face or head, which heals with difficulty and frequently recurs, sometimes alternating with or accompanied by ophthalmia of the same side, should lead to examinations of the lungs, where pneumonia of the apex of the same side is often present.

(4.) Certain sympathetic disturbances of one side of the face or head, namely, frequent changes in color from flushing to pallor, transiatory circumscribed erythema of the cheek or temple, always on the same side, the easy production of Trousseau's maculæ, which also accompany meningitis, cerebral tumors, and other diseases to be excluded, often indicate pneumonia of the apex of the same side.

In several cases of brain tumors which the author has lately observed, he has found lung infiltration on the same side with the brain tubercles.

(5.) Intermittent sympathetic neuroses affecting one side of the head, characterized by elevation of the temperature of the skin of the affected side, are often observed in children with lung infiltrations of the same side. The red and hot ear presents the same phenomena as those noticed in animals after cutting the sympathetic of one side.

(6.) Finally, neuralgias of the trigeminus, oculomotorius, and vagus occurred and disappeared during the process in the lung of the same side in such a manner that no certain relation between the two could be determined.

All the above mentioned symptoms and appearances were observed by the author in a large number of cases; so he concludes that the probability of simple coincidence is inadmissible.

The ways by which these concomitant affections are conducted are through both the lymphatics and the sympathetic.

The author intends to give the more minute details in an extended work, and wished by these preliminary communications to lay claims to priority in such valuable and interesting observations.—*Dr. Hayden, in Boston Journal,* Aug 10, 1876.

*Diphtheria Treated with Sulphate of Iron.*—Sabata (*Annali di Chimica Applicata alla Medicina,* October, 1875,) uses a gargle of five grammes (77⅙ grains) of sulphate of iron in 100 grammes

(3½ ozs. nearly) of distilled water, to which are added 25 drops of sulphuric acid. At the same time he touches the diphtheritic patches with a somewhat stronger solution (5 grammes of sulphate of iron, 70–80 grammes of water, and 25 drops of sulphuric acid.) In addition to these, he orders internally hyposulphite of soda and tannate of quinine. Under this treatment, his statistics show, in the course of a severe epidemic, a death-rate of 12.5 per cent.—*Dublin Med. Jour.*, June, 1876.

*Case of Constitutional Hemorrhage.* By P. W. Douglas, M. D., Dublin, Ga.

I was called to a lad 11 years old, in consultation with my friend, Dr. Carter. Found boy pale, cadevorous, and pulseless. The doctor remarked that he had used various caustic applications, nitrate of silver and persulphate of iron especially, and had done so for thirty-six hours without any abatement of the bleeding. He deemed it unnecessary, as I myself did, to enter into any consultation, as the diagnosis was too apparent, simply giving me his line of treatment, which, in addition to what has been already mentioned, consisted in laudanum and digitalis to quiet him, and reduce the heart's action, which was now alarmingly frequent. We continued the application of the Monsell salt, applied on the finger, and held firmly to the wound in the roof of the mouth, the sub-palatine artery was cut by a piece of glass bottle while eating. In about an hour and a half after my arrival, the parents stated to me that this was the fifth time that he had reached the verge of death from hemorrhage, caused by the slightest wound. One time an abscess was lanced, at another a tooth was pulled, at another a cut on the finger, etc. I at once decided to give him fl. ext. ergot, which was readily assented to by Dr. Carter. We gave him one drachm, and in thirty minutes the bloody water that had been oozing from his mouth in puddles on the floor ceased, the pulse increased in volume and became strong, a general warmth came to the entire surface, and the improved symptoms were so visible that hopes for his recovery were at once established. We gave him some laudanum with the next dose of ergot in an hour, and he passed a good night, and went on to recover without an untoward symptom.

*Diphtheritic Sore-throat.*—Dr. Lolli, of Florence, Italy, states that he has adopted the following treatment in this affection with the best results ; 1. Never cauterize the throat or abstract blood ; abstain from purgatives and emetics, unless in very exceptional cases. 2. Nourish the patient according to his appetie, but let the food be light and easily assimilated. 3. Keep up the functions of the skin from the very commencement of the disease till the local, or still better the general, symptoms allow you to judge that the morbid process is extinct. (Great stress is laid on this point.) 4. For local application as well as for internal use, the author strongly recommends the following "anti-diphtheritic mixture :"

| | |
|---|---|
| Boiling water | oz. vj–xx |
| Liquid sesquichloride of iron | *m* xx–dr. j |
| Carbolic acid | gr. iij–xx |
| Red honey | oz vj |

This can be used internally and as a gargle every two hours, one or two spoonfuls being a dose. This treatment gives a mortality less than two per cent ; average duration of attack, eight or ten days.—*Repetoir Faliciense.*

*Treatment of Fractures.*—Dr. Schwab, of Wurzburg, states that for the past twelve years he has used the following method exclusively and with complete success in the treatment of all fractures of the extremities. Contrary to Professor Hamilton, he says no shortening ever followed : "Take the whites of six to eight eggs, an old linen sheet, from which a bandage of scultetus can be cut, a piece of pasteboard, which is always at hand in the cover of an old book, and a roller bandage from three to four yards in length. Saturate the bandage of scultetus with the albumen and carefully apply, allowing the edges to slightly overlap. This bandage should reach to the joints above and below the fracture. Then saturate the pasteboard with the albumen, adjust it to the part, and secure with the roller. The limb is kept in proper position by means of small bran-bags or cushions of straw."

*Bromide of Lithium.*—Dr. Rouband writes in the *Revue de Therapeutique* as follows of this drug: "I. Bromide of lithium is a drug which has a two-fold action. 2. It possesses in a high degree the lithontriptic qualities which are universally recognized in the salts of lithia. 3. It affects reflex sensibility in a more energetic manner than the other bromides, without the unpleasant effects on the heart which the bromide of potassium has. 4. Consequently it takes its place in the first rank of antilithic and sedative drugs, and its action is especially valuable in cases of the uric-acid diathesis which are accompanied by painful phenomena."

*Treatment of Superficial Varices.*—Cazin (*La France Med.*, December 20, 1875,) referring to the treatment of these cases, and the avoidance of phlebitis of pyæmia, recommends the following proceeding; An incision, three centimetres long, is made parallel to the vein, and at a distance of one centimetre from it. At the two extremities of this incision two others are made transversely towards the vein, and reaching to it. This flap is dissected up, and the vein is isolated by a blunt instrument. The flap is next passed beneath the vein and replaced in its original position, the vein remaining thoroughly isolated without any ligature having been used.—*Dublin Med. Journal*, June, 1876.

*Liquid Bismuth or Elixir of Bismuth.*—The following is the formula adopted by the American Pharmaceutical Association:

Ammonio-citrate of bismuth............256 grains.
Warm distilled water..................... 1 fluid ounce.
Water of ammonia sufficient to neutralize.
Simple elixir................................ 15 fluid ounces.

Mix and filter.

*Dr. Edward Smith*, author of an excellent work on "Foods," thinks that condensed milk is not a suitable food as a substitute for pure milk for infants. It is more fattening, but less nourishing, and greatly reduces the child's power of resisting diseases. Dr. Smith states that children brought up on impure London-fed cow's milk will resist an attack of acute disease better than children fed on condensed milk.

*Acne.*—Every evening for four evenings the face is smeared over with black soap, and in the morning this is removed with tepid water. Then during four consecutive days a warm vapor douche is directed upon the face. The black soap is then resumed, followed by the douches; and so on alternately for a period of six weeks, when the malady, if not entirely removed, is very sensibly mitigated.

*Nitrite of Amyl in Hiccough.*—Dr. Simon, of Chicago, reports in the *Chicago Medical Journal and Examiner* that a patient who had hiccoughed for twenty-six hours, and been subjected to various treatments without success, was relieved almost instantaneously by inhaling three drops of the amyl.

*Incontinence of Urine.*—Mr. Brenchley writes to the *Pactitioner* that he has seldom seen much good done in the above disease by belladonna, iron, or bromide of potassium, but has met with much success, with the following combination of ergot and iron:

R. Tinct. ergotæ................................*mx.*
Tinct. ferri perchloridi......... ........*mv.*
Spts. chloroformi... .......................*mv.*
Infus. quassiæ........... .........ad. oz. j. ter die sum.

*PostPartum Hemorrhages.*—A very simple and efficient method of arousing an inert uterus to contraction is to dip the end of a towel in cold water and smartly slap the hypogastrium. Prof. Isaac E. Taylor, with whom the plan of treatment originated, says that he never saw it fail when properly carried out.—*Med. Record.*

*To Remove Foreign Substances from the Ear.*—Place the patient under chloroform, with the ear affected downwards, and syringe from below. Pull the auricle backwards and upwards (by this means the external auditory meatus is made into a straight tube) and apply the nozzle of the syringe to the upper wall of the passage. The water is then gently forced behind the obstruction; the foreign body is loosened, and its own weight will cause it to fall out of the ear.

*Pills for Obstinate Neuralgia.*—The *Bordeaux Medical* gives the following formula for obstinate neuralgia, especially ileo-lumbar neuralgia:

R. Valerianate of ammonia,
Quinine............ ........ .... ......aa gr. xxx.

Make into twenty pills, and take from two to ten of them each day, increasing one pill per diem. After taking these pills for ten days, suspend their use for five days.

R. Sulphate of iron........................ } aa oz. ijss;

Carbonate of potash.................... }

Mix well and evaporate sufficiently to divide into five hundred pills. Commence with one three times a day, after each meal. Every three days increase the dose by one pill, until six are taken at a dose. If Trommer's Extract of Malt be taken with the above, few cases of simple Chlorosis will resist them.

(Improved Blaud's pills.)

—*Louisville Medical News.*

R. Tinct. cinch. comp.................. }
Tinct. guaiac comp.................. } aa oz. j;
Mel. despumat......................... }
Potass. chlorat............................sc. iv.
Aquæ.......................................oz. iv.

M. S. Use as a gargle every half hour for a few minutes at a time, swallowing a small portion, in Tonsillitis. — *Louisville Medical News.*

*Hydrastin Canadensis in Gleet.*—A cotemporary says: We have found the injection of hydrastin canadensis very efficacious in gleet, the following formula being used:

R. Hydrastin canadensis..................drs. ij
Liq. morphiæ mur.....................drs. ij
Aquæ.......................................
Mucilag. acaciæ.........................aa oz. iss M.

*Remedy for Dandruff.*—A French physician recommends to apply a solution of chloral hydrate containing 5 per cent. of the latter, by rubbing from ½ to 1 oz. into the scalp by means of a sponge, and repeating it every morning. A slight burning sensation and reddening of the scalp occurs, disappearing after two minutes. If the hair had fallen off in consequence of the dandruff, it will be renewed in about a month.—*Apoth. Ztg.*, No. 25.

*Goddard's Cosmetic Lotion.*—

R. Tinct. benzoini.................................f. dr. ij.

Hydrarg-chloridi corrosivi....... ..................gr. vi.
Aquæ rosæ............ . . . ..............................f. oz. vi.

Fiat mistura.

A valuable cosmetic lotion, but must be used with caution, as it irritates some skins.

*Extract of Beef.*—Bouchardat warns against the incautious use of this extract, under the mistaken notion that an increase of dose will be followed by a corresponding increase of benefit. Both he and Stuart Cooper have shown that large doses of the extract are quite injurious. He further asserts that it cannot at all be compared to meat-juice (expressed in the cold from raw meat) as a strength giver.—*Archiv for Pharm.*

*Spina Bifida.*—In the *Annali Universali di Medicina* for April Dr. Parona narrates a case of cervical spina bifida which he successfully treated by Prof. Rizzoli's method of applying a constrictive forceps at the base of the tumor. It makes the fourth successful case recorded.—*Lond. Lancet*, July 22, '76.

*Mist. Biniodidi.*—

R. Hydrarg. Bichlor..................................gr. 1
Potass. Iod.......................... ..................dr. 2
Tinct. Gent. Co...............................fl. oz. 2

[Tr. Cinch. is less proper, because some of the alkaloids are precipitated with iodohydrargyrate of potassium.]

M. Teaspoonful.

*Porrigo Decalvans.*—In this disease, Dr. Lespian, of Paris, recommends the following formula:

R. Tannic acid..................................grs. xv.
Tinct. iodin...................................drs. ijss.
Glycerine............................ ... .... drs. v. M.

Apply to the part twice daily.

*Pathognomonic Symptom of Cerebro-spinal Meningitis.* — Dr. Hayden, of Dublin, a competent authority, states that he never saw a case of cerebro-spinal meningitis unattended by pains in the calves of the legs, and he should make a presumptive diagnosis from the presence of that symptom alone.

*Bicarbonate of Soda in Suppression of Urine.*—Mark Lemon, M.B., has used bicarbonate of soda in half teaspoonful doses thrice daily with excellent effect, in renal cases attended with suppression of urine. In connection with warm bathing, he has found nothing so useful as it is in the class of cases mentioned. —*New Remedies.*

*Treatment of Capillary Nævus.*—Mr. Bradley states, in the *British Medical Journal*, that he was induced to try the effect of treating the ordinary capillary nævus, or mother's mark, by tattooing with carbolic acid. In the only case in which he has tried it, the port wine stains disappeared in about three weeks. He recommends it for further trial.

*Antidote to Strychnia.*—The East Indian physicians recommend nicotia as the surest antidote, which is given in exceedingly small quantities in sherry several times a day. In default of nicotia, a decoction of tobacco leaves (one half ounce to a pint) is given.—*Druggist Circular and Chemical Gazette.*

*Mist. Diarrhœa Ruschenberger, B. & C. H.*—

R. Tinct. Opii.................................
  " Camph..........................
  " Capsici......................... . } aa p. e.
  " Menth. Pip.......................
  " Rhei Arom .......................

M. 30 to 40 drops in water.

*Poisoning by Belladonna and Cure.*—Dr. H. L. Horton, of Morrisania, N. Y., reports the case of a boy two and a half years old, who swallowed forty-five grains extract belladonna, who was cured of its effects by four teaspoonfuls of laudanum.—*Druggist Circular and Chemical Gazette.*

*For Burns.*—

R. Glycerine.........................................oz. v
  White of egg.......................................oz. iv
  Tincture of arnica.................................oz. iij.

Mix the glycerine and white of egg immediately in a mortar, and then add gradually the arnica. Apply freely on linen cloths night and morning, having previously washed the burn with castile suds.

*Chloral Plaster.*—For neuralgia, rheumatic pains, etc., use the ordinary emplastrum roborans, and powder it with the chloral. Apply the plaster to the affected part and leave it from twenty-four to forty-eight hours. When taken off the skin is found studded with vesicles; these are to be picked with a pin, followed by a dressing with simple ointment. The pain vanishes long before the vesicles are dried up.

*Syrup of Salicylic Acid.*—In giving this acid, the annexed formula, for a syrup, has been suggested:

R. Salicylic acid........ ............ .............drs. ss.
Oil of sweet almonds ..........................drs. x.
Gum arabic.....................................drs. x.
Syrup cf almonds.......... ..... ................drs. xij.
Orange flower water..........................drs. xij.— M.

*Chlorate of Potassium in Chronic Gastro-Intestinal Catarrh.*—S. C. Dumm, M.D., used chlorate of potassium in a case of chronic gastro-intestinal catarrh, attended with inflammation of the bile duct, with excellent results. Ten grains were given every three hours, dissolved in water. After the use of the remedy for one day, pain in the stomach ceased, the appetite improved, vomiting stopped, and the patient recovered her health quickly.—*New Remedies.*

*Toothache Remedy.*—Mr. C. A. Guild writes to the *Clinic:* "I have found collodion mixed with enough carbolic acid to form a jelly-like mass to be an excellent remedy for toothache. About equal parts will form a 'stiff' jelly, which may be taken on the end of a pine stick and placed in the cavity of the aching tooth. The pain will be relieved almost instantly if it depends on an exposed nerve. I have found this the most reliable and convenient remedy I ever tried."

*Phosphorus.*—The solution of phosphorus in alcohol generally takes from 12 to 24 hours, and the alcohol has moreover to be kept warm all the time. It is, therefore, proposed to use glycerin as a solvent. Phosphorus dissolves quite readily by shaking for several minutes with warm glycerin; an addition of warm alcohol will prevent any deposit of phosphorus in cooling —*Apoth. Teit.* 1875

*Cincho-Quinine, Strychnine and Iron.*—The following is an elegant combination of these valuable remedies, and has been found to produce the most favorable results:

R. Cincho-quinine.............................. 64 grs.
Strychnine.... ................................ 4 grs.
Tr. ferri mur...................................drs. xviij.
Syrup......... .. ...............................q. s.—M.

Triturate the cincho-quinine and strychnine in a glass mortar, adding the tincture of iron gradually and a few drops of nitric acid if necessary, until they are dissolved; filter, and add syrup to make the finished preparation to measure one pint.

*Nerve Stretching in Tetanus.*—In a case of tetanus which occurred in the Montreal General Hospital, Dr. Drake cut down upon the sciatic nerve and stretched it. The patient was then put upon chloral hydrate and calabar bean. The operation seemed at first to afford considerable relief to the patient, but after a time the spasms returned and he ultimately died of lock-jaw.

*Poisoning by Carbolic Acid.*—As this acid is now so extensively used, it may be of some importance to make known the antidotes which have been proposed. M. Ferrand advises the following: White sugar, 15 parts; water, 40 parts; quicklime, 5 parts—forming a saccharate of lime.—*Lancet.*

*Antidote to Strychnia.*—The East Indian physicians recommend nicotia as the surest antidote, which is given in exceedingly small quantities in sherry several times a day. In default of nicotia, a decoction of tobacco leaves (½ ounce to a pint) is given.—*Archiv for Pharm.*

*Scrofulous Sores.*—A Scotch clergyman states in the *Edinburgh Medical Journal* that three grains of corrosive sublimate in a pint of whisky constitutes an almost infallible remedy in scrofulous sores or runnings. A rag dipped in this twice or thrice a day should be kept on the ulcers until healed.

*For Whooping Cough.*—The latest remedy for whooping cough, is Spanish chestnut leaves. It is given in the form of infusion of the leaves, 1 to 2 ounces to the pint. Dose, one to two tablespoonfuls every two or three hours.

